# Cardiac myocyte intrinsic contractility and calcium handling deficits underlie heart organ dysfunction in murine cancer cachexia

**DOI:** 10.1038/s41598-021-02688-z

**Published:** 2021-12-08

**Authors:** Michelle L. Law, Joseph M. Metzger

**Affiliations:** 1 Nutrition Sciences Division, Department of Human Sciences and Design, Robbins College of Health and Human Sciences, Baylor University, Waco, TX USA; 2 Department of Integrative Biology and Physiology, University of Minnesota Medical School, Minneapolis, MN USA

**Keywords:** Cancer, Cell biology, Cardiology, Oncology

## Abstract

Cachexia is a muscle wasting syndrome occurring in many advanced cancer patients. Cachexia significantly increases cancer morbidity and mortality. Cardiac atrophy and contractility deficits have been observed in patients and in animal models with cancer cachexia, which may contribute to cachexia pathophysiology. However, underlying contributors to decreased in vivo cardiac contractility are not well understood. In this study, we sought to distinguish heart-intrinsic changes from systemic factors contributing to cachexia-associated cardiac dysfunction. We hypothesized that isolated heart and cardiac myocyte functional deficits underlie in vivo contractile dysfunction. To test this hypothesis, isolated heart and cardiac myocyte function was measured in the colon-26 adenocarcinoma murine model of cachexia. Ex vivo perfused hearts from cachectic animals exhibited marked contraction and relaxation deficits during basal and pacing conditions. Isolated myocytes displayed significantly decreased peak contraction and relaxation rates, which was accompanied by decreased peak calcium and decay rates. This study uncovers significant organ and cellular-level functional deficits in cachectic hearts outside of the catabolic in vivo environment, which is explained in part by impaired calcium cycling. These data provide insight into physiological mechanisms of cardiomyopathy in cachexia, which is critical for the ultimate development of effective treatments for patients.

## Introduction

Cancer cachexia is a syndrome of body weight loss and muscle wasting present in an estimated 30% of all cancer patients^1^. Patients with cancers of the gastrointestinal tract, lung, and liver are most affected, with an estimated risk for developing cachexia between 70 and 90%^1^. Cachexia causes severe weakness and fatigue and leads to progressive functional impairment and diminished quality of life^2^, eventually causing approximately 20% of cancer deaths^3^. Cachexia decreases tolerability and effectiveness of cancer therapies^4^ and increases both cancer-related complications and medical costs^5,6^. Cachexia is caused by a complex interplay of tumor- and host-derived factors leading to systemic inflammation, metabolic aberrations, and increased energy expenditure, which is often complicated by decreased appetite and energy intake^7,8^. There are no approved therapies to prevent or treat cancer cachexia^9^.

In addition to significant skeletal muscle wasting, cardiac atrophy also occurs in humans and animal models with cachexia^10^. This is accompanied by impaired in vivo contractile function measured by echocardiography in humans and both echocardiography and invasive hemodynamics in rodents^10–13^. Contractile dysfunction occurs independently of decreased food intake in mice^11^, implicating direct tumor effects on the heart. Increased inflammatory signaling leading to proteolytic degradation of sarcomeric proteins^11,14^ and upregulated autophagy^15^ have been proposed as potential mechanisms underlying ventricular atrophy. Fetal gene reactivation^14^, mitochondrial morphological^11^ and functional changes^16,17^, and aberrant fatty acid oxidation^18^ may contribute to both decreased cardiac mass and function, although more work is needed to understand causal relationships.

Complicating the study of cardiac function in vivo is the inability to identify specific functional deficits intrinsic to the cardiac muscle outside of the catabolic and inflammatory milieu of the cachectic animal. Tumor- and host-derived cytokines and metabolic and hormonal changes act systemically, altering function of numerous tissues in the body^19^. A limited number of studies have tested approved therapies for cardiovascular disease in cachectic animals, including ACE inhibitors, beta-blockers, and aldosterone antagonists. These studies have yielded inconsistent results related to cardiac function^10,20^. This inconsistency may be attributable to the use of different models and severities of cachexia. In addition, an incomplete understanding of the development and progression of cachexia-induced cardiomyopathy and lack of knowledge regarding how organ- and cellular-level functional deficits contribute to in vivo dysfunction makes identification of appropriate therapeutic strategies challenging. Understanding underlying physiological mechanisms contributing to cachexia-induced cardiomyopathy and the similarities and differences compared to “classical” heart failure will provide necessary insight into optimal therapeutic targets. Effectively treating the heart in cachexia is imperative because cardiac functional deficits can directly contribute to fatigue and muscle wasting^21^. As a result, cardiac dysfunction in cachexia may be a contributor to cachexia pathophysiology, creating a vicious cycle of muscle wasting, fatigue, and cardiac insufficiency^22^.

The objective of this study was to distinguish potential heart organ and cellular intrinsic changes from systemic factors underlying cachexia-mediated cardiomyopathy. To accomplish this, we investigated the function of isolated hearts and cardiac myocytes from mice with tumor-induced cachexia to understand underlying physiological mechanisms of in vivo cardiac dysfunction. We hypothesized that contractility deficits in ex vivo hearts and isolated cardiac myocytes underlie in vivo cardiac dysfunction. Additionally, we hypothesized that impaired cell intrinsic calcium cycling contributes to functional deficits.

## Methods

### Cell culture

Colon-26 adenocarcinoma (C26) cells (Division of Cancer Treatment and Diagnosis Tumor Repository, National Cancer Institute) were cultured in RPMI 1640 + L-glutamine medium (Gibco, Thermo Fisher Scientific) supplemented with 5% (v/v) fetal bovine serum (FBS) and 1% (v/v) Penicillin–Streptomycin (10,000 U/ml) at 37 °C with 5% CO_2_. At ~ 75% confluence, cells were washed, trypsinized, centrifuged, and resuspended in sterile PBS at a concentration of 1.0 × 10^7^ cells/ml immediately prior to mouse inoculation.

### Animals

Male CD2F1 mice with colon-26 adenocarcinoma tumors were used in all experiments. This model recapitulates numerous aspects of the human condition, including body and muscle wasting, decreased strength, fatigue, systemic inflammation, insulin resistance and impaired macronutrient metabolism, and anorexia^23–25^. Male CD2F1 mice were obtained by breeding female BALB/c and male DBA2 mice from Charles River Laboratories (Wilmington, MA). Males were utilized in this study as they exhibit a highly consistent and robust cachectic phenotype compared with females^15,20,24,26^. The number of animals used in each experiment was determined from previously published work^27,28^. A total of 65 animals were used in the experiments. Eight animals were not used for the following reasons: (1) three met humane endpoint criteria prior to the end of the study; (2) one anesthetized animal stopped breathing prior to heart excision in the physiology experiments; and (3) four hearts had nicked or torn aortas preventing their use in the physiology experiments. Due to the time-intensive nature of the physiology experiments, all 73 animals were not injected with tumor cells or control and used at a single time point. Mice were bred and put through the study protocol for the various experiments in cohorts as detailed below.

**Figure 1:**
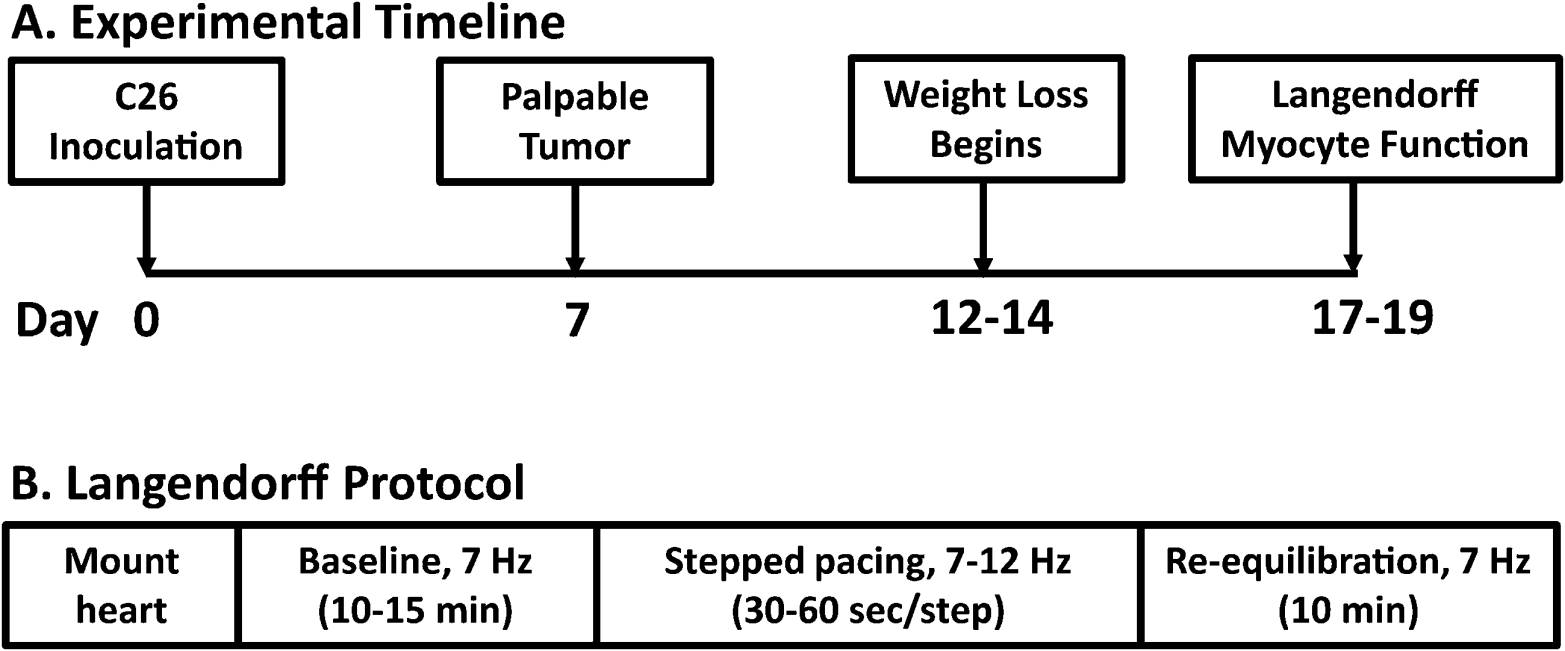
Experimental timelines. (**A**) Timeline for the animal study. All experiments were completed 17–19 days after C26 cell inoculation, when mice had significant weight loss and muscle wasting. (**B**) Langendorff protocol. Hearts were mounted and equilibrated, and baseline pacing data were collected at 7 Hz. A subset of hearts underwent a stepped pacing stress protocol, with a pacing increase of 1 Hz every 30–60 s up to 12 Hz. Hearts were then re-equilibrated at 7 Hz to conclude the experiment.

Mice were housed on a 12-h light–dark cycle and given ad libitum access to standard rodent chow and water. When mice reached ~ 8 weeks of age (20–25 g), each cage of mice was randomly assigned to the Tumor group or Control group. Mice in the Tumor group received 1.0 × 10^6^ C26 cells in 100 µl PBS injected subcutaneously in the left flank region. The Control group received an equal volume of PBS without C26 cells. C26 cells grow into a solid, non-metastatic tumor that is palpable after ~ 7 days and begin to induce significant weight loss by 2 weeks post-inoculation^24^. During the study, body weight was measured periodically to monitor weight loss. At 17–19 days after C26 inoculation, mice were given intraperitoneal injections of heparin (250 IU) and sodium pentobarbital (100 mg/kg). When mice were unresponsive to a toe pinch, the chest was opened and hearts were excised via terminal thoracotomy for use in the experiments described below (Fig. 1A). Tumor, tibialis anterior muscle, and heart weight were collected in a subset of animals. Mice were euthanized at 17–19 days post-tumor cell inoculation because at this time significant body weight and muscle loss has occurred, but humane endpoint criteria have not been met in most animals. Any animals meeting endpoint criteria prior to the 17–19 day time point were euthanized and not included in the study. All procedures for this study were approved by the University of Minnesota Institutional Animal Care and Use Committee. All methods were carried out in accordance with relevant guidelines and regulations. Reporting of procedures and data follow ARRIVE guidelines^29^.

### Langendorff

After excision, hearts were immediately placed in ice-cold, modified Krebs–Henseleit Buffer (KHB; In mmol/L: 118 NaCl, 4.7 KCl, 1.2 MgSO_4_, 1.2 KH_2_PO_4_, 0.5 NaEDTA, 2.5 CaCl_2_, 10 glucose, 25 NaHCO_3_), where excess tissue and atria were removed, aortas cannulated, and ventricles flushed to remove excess blood. Hearts were then mounted on a Langendorff apparatus and retrogradely perfused with oxygenated modified KHB at 37 °C. A balloon catheter attached to a pressure transducer was placed into the left ventricle for isovolumic pressure measurements, and an electrode was positioned on the left ventricle to control pacing frequency^30^. A schematic illustrating the experimental protocol is shown in Fig. 1B. Hearts were paced at 7 Hz for 10–15 min to allow for equilibration and baseline functional measurements. At that time, a subset of hearts underwent a pacing stress protocol in which pacing frequency was increased incrementally from 7 to 12 Hz every 30–60 s (~ 3–5 min total time to reach the maximum frequency). The experiment concluded by re-equilibrating hearts at 7 Hz for ~ 10 min to ensure baseline function was recovered^30^. This pacing protocol is representative of the in vivo environment as the heart rate in unanesthetized mice increased from ~ 480 beats per minute (8 Hz) at rest to ~ 720 beats per minute (12 Hz) after 5 min of treadmill running^31^. Data were averaged from approximately 10 contraction/relaxation cycles after equilibrium was reached at each pacing frequency.

### Cardiac myocyte isolation and culture

Cardiac myocytes were isolated as previously described^32^. Briefly, hearts were excised, aortas were cannulated and hearts mounted on a modified Langendorff apparatus where they were perfused with oxygenated modified KHB (in mM: 118 NaCl, 4.8 KCl, 25 HEPES, 1.2 KH_2_PO_4_, 1.2 MgSO_4_, 11 glucose, 30 taurine, 10 2,3-butanedione monoxime) with 620 U/ml collagenase type II (Worthington Biochemical, Lakewood, NJ), for about 10 min until the perfusion rate began to increase. At this time, hearts were removed from the Langendorff, atria trimmed off, and ventricles cut into about 8 pieces. Ventricular tissue was gently titurated to release individual myocytes. Myocytes were centrifuged and resuspended in stop buffer [modified KHB with 2.5% bovine serum albumin (w/v), 5% FBS (v/v), and 0.1 mM CaCl_2_]. Calcium was added back to a concentration of 1.8 mM in a stepwise fashion. Myocytes were then plated on laminin-coated, glass coverslips and cultured in M199 medium (Gibco, Thermo Fisher Scientific) containing 5% FBS at 37 °C and 5% CO_2_ for 1 h prior to physiological experiments.

### Cardiac myocyte dimensions and sarcomere length measurements

Cardiac myocyte dimensions and contractile and relaxation function were measured using the IonOptix Calcium and Contractility System (Westwood, MA, Ionoptix.com). Briefly, glass coverslips containing isolated myocytes were mounted onto a stimulation chamber and bathed in Tyrode’s solution warmed to 30 °C. Length and width were measured with the Edge Detection feature in quiescent myocytes. Myocytes were then and stimulated at 25 V with 0.5 Hz pacing frequency and the Sarcomere Length feature was used to measure contractility and relaxation. Cells were visualized with an inverted microscope (Nikon Eclipse TE2000-U) fitted with a 40X objective. Sarcomere length data was collected at 1000 Hz, and approximately 10 contractile cycles were averaged for each cell. Data were analyzed by Fast Fourier Transform using IonWizard Software (IonOptix, Westwood, MA, Ionoptix.com), and ~ 15 myocytes were analyzed from each of 3 mice per group. These numbers were chosen to obtain a representative sampling of myocytes from each heart, and to ensure functional trends and inter-myocyte variability were similar between mice.

### Calcium transient measurements

Isolated cardiac myocytes were incubated at room temperature, in the dark, with 2 µM of the calcium indicator dye Fura-2AM (AbCam, Cambridge, MA) for 10 min, followed by 20 min of de-esterification. Calcium transients were measured using the IonOptix Calcium and Contractility System (Ionoptix.com), with the µ-stepper switch to measure 360 nm and 380 nm wavelengths. Myocytes were bathed in 30 °C Tyrode’s solution and paced at 25 V and 0.5 Hz. The 360:380 nm ratio was collected to measure the relative change in intracellular calcium concentration during the contractile cycle. The excitation wavelength of 360 nm measures both calcium-bound and unbound Fura-2AM, whereas 380 nm measures unbound Fura-2AM. The 380 nm wavelength is measured at 1000 Hz, and the 360 nm wavelength is measured every 10 s to account for photobleaching or loss of Fura-2AM from the cell. Approximately 10 transients were averaged per cell and analyzed using IonWizard Software (IonOptix, Westwood, MA), and ~ 15 myocytes from each of 3 mice were analyzed per group.

### Histology

Hearts were excised, rinsed with PBS, and cross-sectional slices were obtained from the center of the ventricles. Heart slices were embedded in OCT and frozen in liquid nitrogen cooled isopentane. Heart slices were sectioned at a thickness of 7 µm and Sirius red/fast green staining was performed by the Lillehei Heart Institute Histology Core Laboratory (University of Minnesota, Minneapolis, MN). Stained sections were imaged using a Nikon Eclipse E600 microscope. Images were stitched together with the pairwise stitching plugin^33^ in Fiji^34^ and the percentage of red-stained cross-sectional area was quantified.

### Statistical analysis

Data were analyzed using SigmaPlot 14.0 (Systat Software, Inc, San Jose, CA). The Shapiro–Wilk test was conducted to determine normality of the data. For most experiments, normally distributed data were analyzed using a two-tailed t-test, and non-normally distributed data were analyzed using the Mann–Whitney Rank Sum test to measure differences between the Control and Tumor groups. For the Langendorff pacing experiment, two-way repeated measures ANOVA was used to determine the effect of tumor, pacing frequency, and tumor by frequency interactions. The Bonferroni t-test for multiple comparisons was used to determine differences between the Control and Tumor groups at each pacing frequency. *P* < 0.05 was considered statistically significant.

## Results

### Body and tissue weight, and cardiac myocyte dimensions

At 17–19 days post-C26 cell inoculation, mice with tumors (Tumor group) had significantly lower body weight and muscle mass compared to mice without tumors (Control group) when expressed both as absolute mass and as a percentage of body weight (Table 1). The extent of body mass and muscle wasting is similar to previously published studies using this model^24,26^. Absolute ventricular mass was decreased by 14% in mice with tumors compared to control (*P* < 0.01), and statistical significance was maintained when normalized to tibia length (*P* < 0.01). However, ventricle mass as a percentage of body weight was similar between Tumor and Control groups, suggesting cardiac atrophy does not occur at a greater rate than whole body wasting. This is in contrast to skeletal muscle, which shows a greater extent of wasting compared to whole body wasting, as indicated by decreased TA mass when expressed as a percentage of body weight (Table 1). Isolated cardiac myocyte width was decreased by 26% in the Tumor group compared to the Control group (*P* < 0.001), without a change in length (Table 1). Decreased myocyte width suggests that smaller cardiac myocyte size and decreased number of myofibrils is contributing to decreased cardiac mass, which is consistent with previous findings of decreased myofilament protein expression^11,14^. Although we cannot exclude a possible decrease in cell number as well, many studies point to proteolysis more than cell death as the major physiological contributor to cachexia-induced muscle atrophy^26,35^. Decreased width in the absence of increased length suggests a condition of cardiac atrophy more than a dilated phenotype.

**Table 1:** Body and tissue weights, and cardiac myocyte dimensions of mice with and without tumors.

	Absolute measurement		Percent body weight^a^	
	Control	Tumor	Control	Tumor
Initial Body Weight (g)	25.3 ± 0.5	25.3 ± 1.0		
Final Body Weight (g)	27.8 ± 0.6	24.4 ± 1.2*		
Body Weight Change (g)	2.5 ± 0.4	− 0.9 ± 0.8**	9.8 ± 1.5	− 3.4 ± 3.3**
Tumor-Free Body Weight Change (g)	2.5 ± 0.4	− 2.4 ± 0.9***	9.8 ± 1.5	− 9.2 ± 3.7 ***
Tibialis Anterior (mg)	50.2 ± 1.7	36.4 ± 3.5**	0.18 ± 0.01	0.15 ± 0.01**
Heart Weight (mg)	126.5 ± 4.0	108.5 ± 3.7**	0.46 ± 0.01	0.45 ± 0.01
Tibia Length (mm)	17.1 ± 0.2	16.9 ± 0.1		
Heart Weight/Tibia Length (mg/mm)	7.4 ± 0.2	6.4 ± 0.2**		
Cardiac Myocyte Length (nm)	119.4 (107.2–135.8)	121.4 (110.1–135.2)		
Cardiac Myocyte Width (nm)	25.6 (19.8–30.4)	18.0 (14.9–21.9)**		
Tumor Weight (g)		1.5 ± 0.1		6.2 ± 0.7

N = 9 mice per group; data are mean ± SEM or median (IQR); *P < 0.05, **P < 0.01, ***P < 0.001 compared to control.

^a^Calculated as: (body weight change/initial body weight) × 100 or (tissue weight/final body weight including tumor) × 100.

### Langendorff-perfused heart function

**Figure 2:**
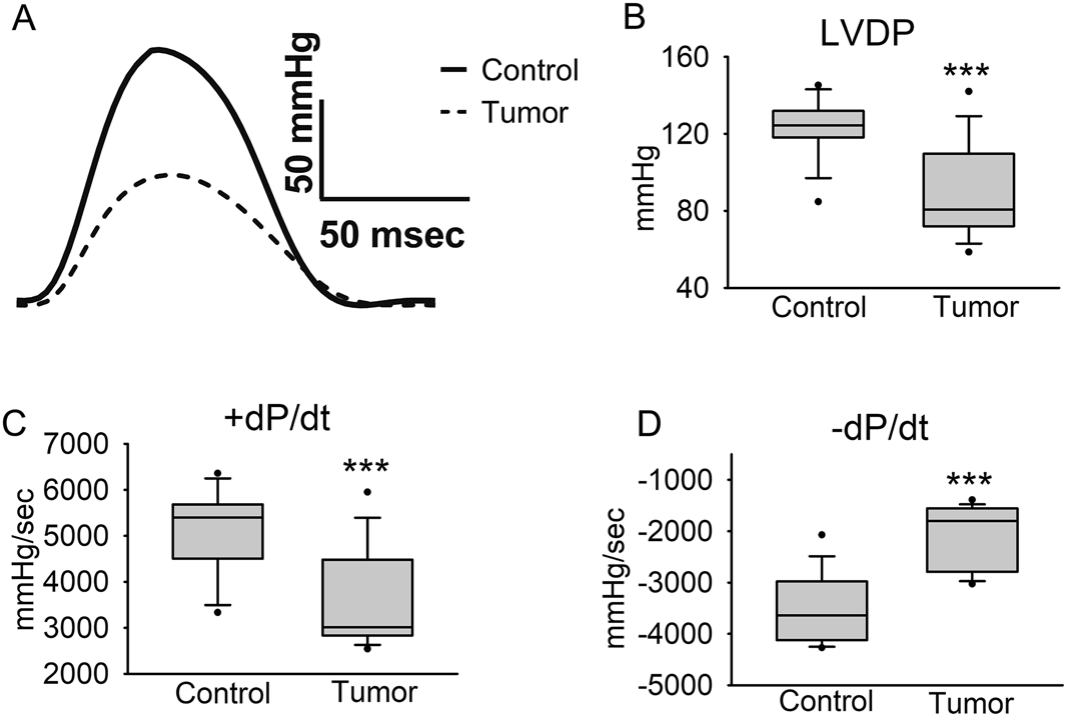
Langendorff heart baseline ex vivo function. Data were collected after hearts were equilibrated at 7 Hz pacing frequency. (**A**) Representative traces showing a complete contraction/relaxation cycle, (**B**) left ventricular developed pressure, (**C**) maximum pressure derivative, and (**D**) minimum pressure derivative. Data are shown as median + / − IQR, N = 15 mice/group, ***P < 0.001 compared to control.

Ex vivo isolated heart function was measured at a baseline pacing frequency of 7 Hz and during a stepped pacing stress protocol as illustrated in Fig. 1B. Representative traces from the Control and Tumor groups are shown in Fig. 2A. Compared to hearts from the Control group, hearts from the Tumor group had significantly decreased left ventricular developed pressure (LVDP) (Fig. 2B) and positive pressure derivative (+ dP/dt) (Fig. 2C), indicating systolic dysfunction. Furthermore, significantly decreased negative pressure derivative (-dP/dt) (Fig. 2D) and prolonged time from peak to 50% pressure fall (Table 2) provides evidence of diastolic dysfunction at baseline pacing frequency. Increased full-duration at half-maximum and peak width (Table 2) demonstrates a prolongation of the entire contractile cycle in hearts from the Tumor group.

**Table 2:** Langendorff-perfused hearts from mice with and without tumors paced at 7 Hz.

	Control	Tumor
End Diastolic Pressure (mmHg)	7.0 ± 0.3	6.8 ± 0.5
Time to 50% Pressure Rise (msec)	24.5 ± 0.4	24.6 ± 0.8
Time to 50% Pressure Fall (msec)	32.6 ± 0.6	36.5 ± 0.8***
Full Duration at Half-Maximum (msec)	57.1 ± 0.6	61.1 ± 1.2**
Peak Width (msec)	99.8 ± 1.9	108.9 ± 2.0**

N = 15 mice per group; data are mean ± SEM; **P < 0.01, ***P < 0.001.

**Figure 3:**
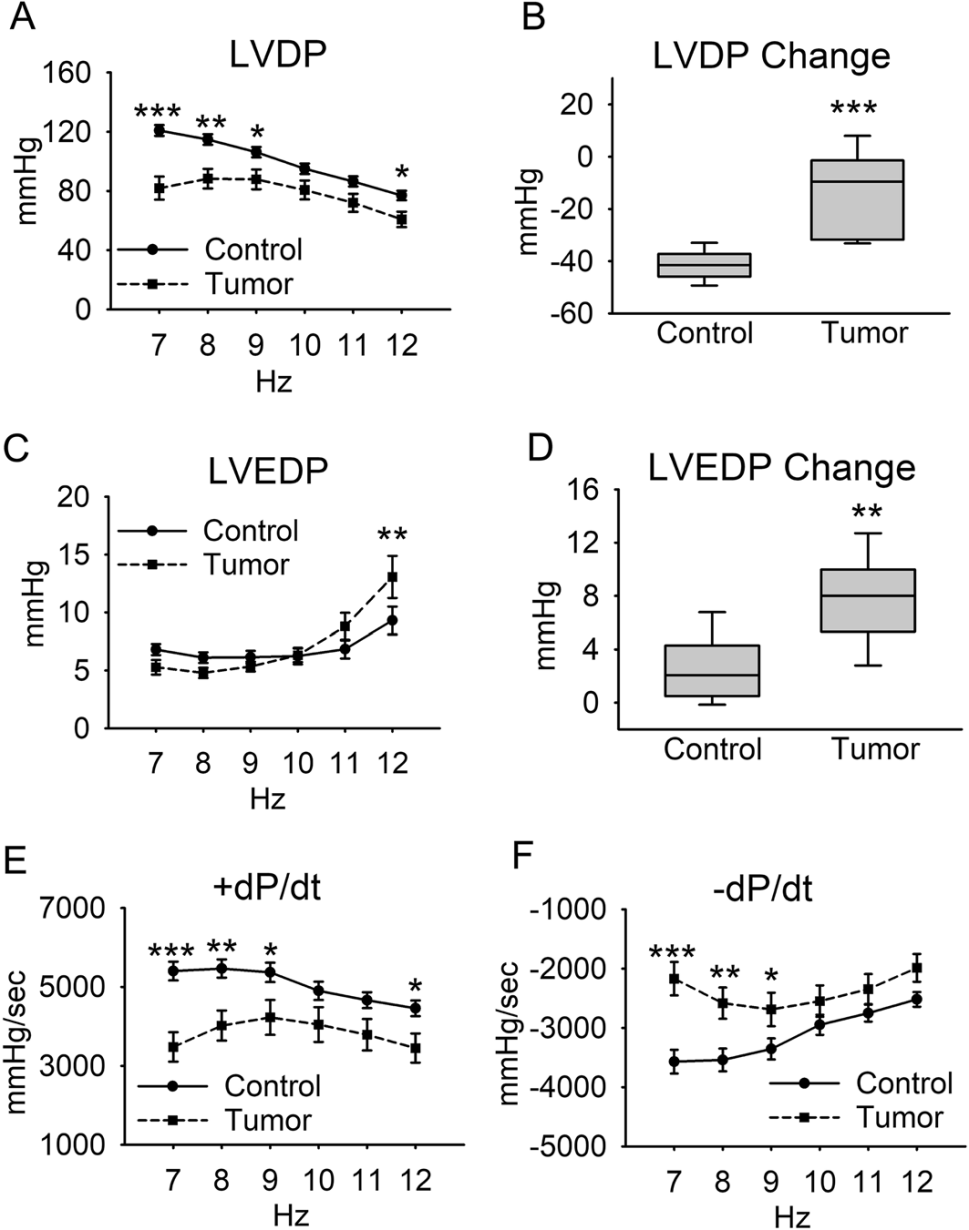
Langendorff heart pacing stress test. Hearts underwent a pacing increase of 1 Hz every 30–60 s from 7 to 12 Hz. (**A**) Left ventricular developed pressure, (**B**) change in left ventricular developed pressure from 7 to 12 Hz, (**C**) left ventricular end diastolic pressure, (**D**) change in left ventricular end diastolic pressure from 7 to 12 Hz, (**E**) maximum pressure derivative, (**F**) minimum pressure derivative. Line graph data are mean + / − SEM, and box plot data are median + / − IQR. N = 6–8 mice/group, *P < 0.05, **P < 0.01, ***P < 0.001.

During stepwise increased pacing from 7 to 12 Hz, the differences in LVDP between the two groups diminished as pacing increased (Fig. 3A). The change in LVDP from 7 to 12 Hz was significantly less negative (Fig. 3B), indicating a differential adaptation to pacing stress in hearts from the Tumor group compared to the Control group. Left ventricular end diastolic pressure (LVEDP) increased significantly in the Tumor group at 12 Hz pacing (Fig. 3C, D), providing evidence of increasing deficits in relaxation function during pacing stress. Both positive (Fig. 3E) and negative (Fig. 3F) pressure derivatives were significantly lower in the Tumor group at lower pacing frequencies, and this difference diminished at higher pacing frequencies. To our knowledge, this is the first measurement of ex vivo Langendorff-perfused heart function in cachectic mice. These experiments provide evidence of significant isolated heart-intrinsic systolic and diastolic dysfunction of the left ventricle, outside of the catabolic and inflammatory environment of the whole animal.

### Fibrosis

**Figure 4:**
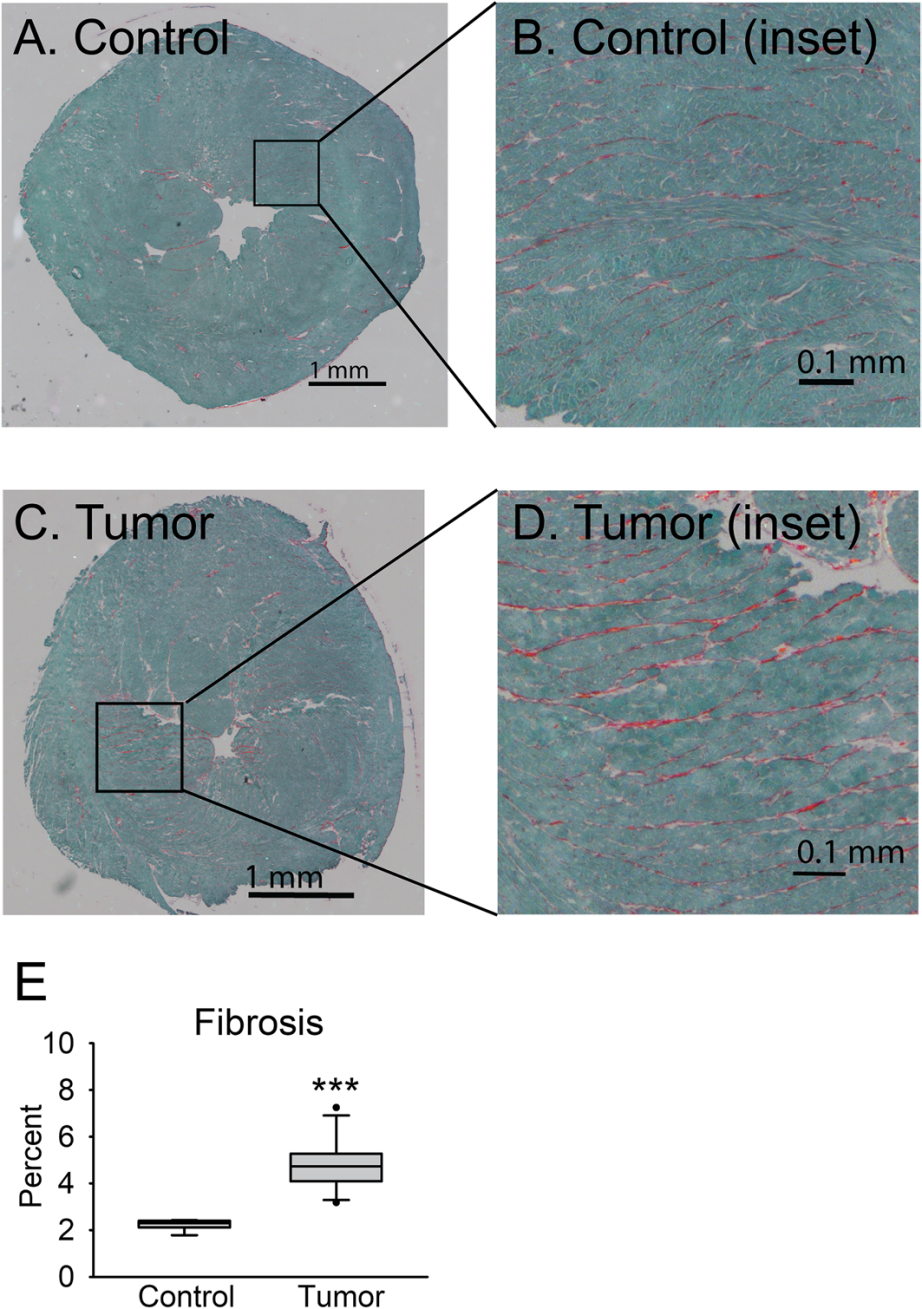
Fibrosis development. Fibrotic area was measured in Sirius red/fast green-stained heart sections as percent of total cross-sectional area stained red. (**A**) Stitched representative image and (**B**) inset for Control group. (**C**) Stitched representative image and (**D**) inset from Tumor group. Calibration bars are 1 mm for (**A**) and (**C**) and 0.1 mm for (**B**) and (**D**). (**E**) Percentage of total fibrotic area is shown as median + / − IQR. N = 6–11 mice/group, ***P < 0.001.

To determine whether fibrosis may be contributing to impaired relaxation function, Sirius red/fast green staining was performed on heart sections. A significant increase in fibrotic tissue was present in the Tumor group (4.8%) compared to the Control group (2.2%) (*P* < 0.001), and fibrosis positive staining was found diffusely spread throughout the ventricles (Fig. 4A–D). Although cardiac fibrosis has been noted in previous studies^10,11^, quantification of fibrotic area in the present work provides evidence of statistically significant increases (Fig. 4E).

### Cardiac myocyte contractility and relaxation

**Figure 5:**
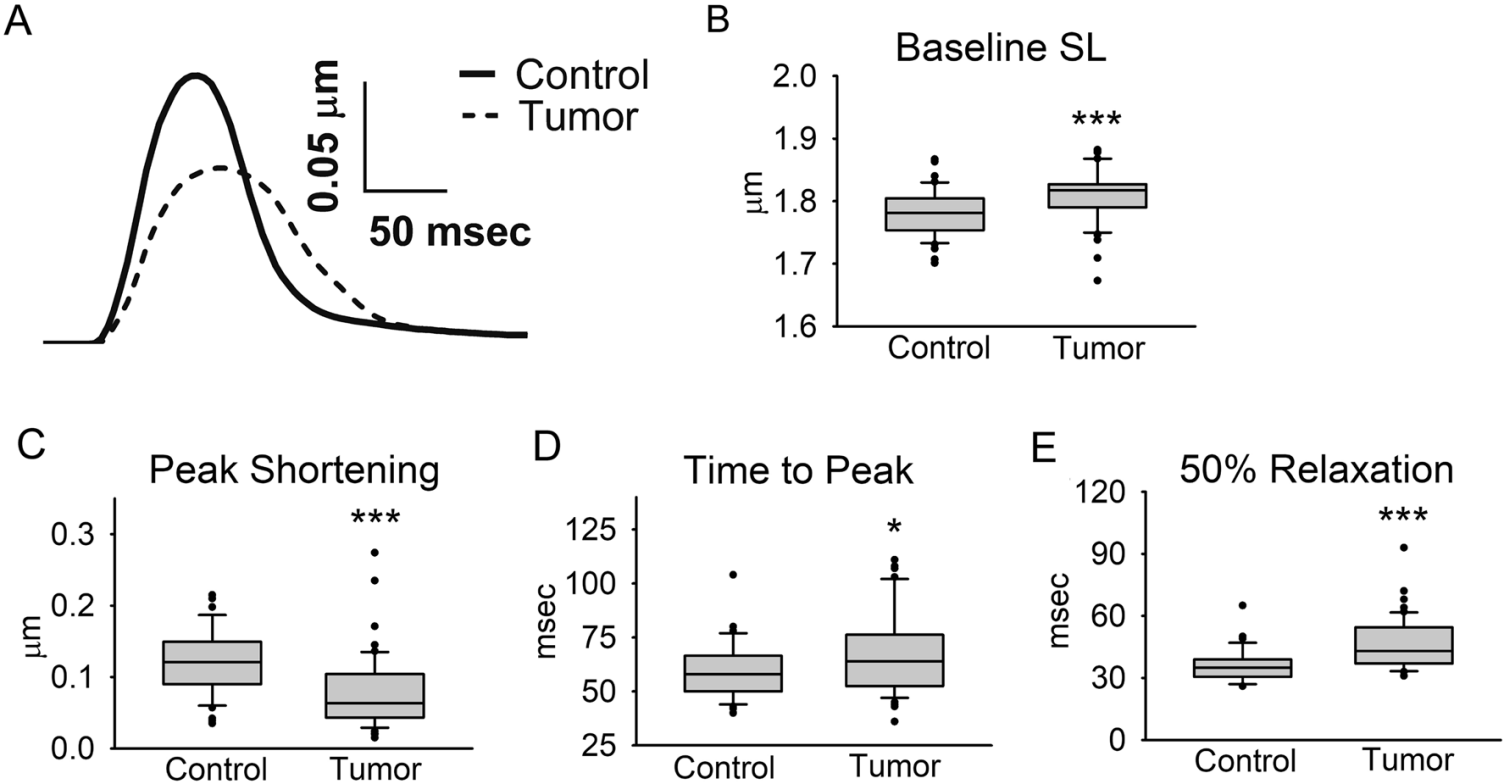
Cardiac myocyte sarcomere length measurements. (**A**) Representative traces of a complete contraction/relaxation cycle, (**B**) baseline sarcomere length, (**C**) peak sarcomere shortening, (**D**) time from baseline to peak sarcomere shortening, (**E**) time from peak to 50% relaxation. Myocytes were paced at 0.5 Hz and 30 °C. N = 49–52 myocytes from 3 mice/group, data are presented as median + / − IQR. *P < 0.05, ***P < 0.001.

Isolated cardiac myocyte contractility and relaxation were measured to determine whether cell-intrinsic functional deficits were contributing to whole heart pump dysfunction. Representative cardiac myocyte sarcomere length contractile traces from the Control and Tumor groups are illustrated in Fig. 5A. Compared to the Control group, cardiac myocytes from the Tumor group had a significant increase in baseline sarcomere length (Fig. 5B), which may indicate decreased diastolic tone. Cachectic myocytes also exhibited decreased contractility, indicated by a 35% decrease in peak sarcomere shortening (*P* < 0.001) (Fig. 5C). Moreover, the time from baseline to peak shortening (Fig. 5D) and time from peak to 50% relaxation (Fig. 5E) were significantly increased in cardiac myocytes from the Tumor group. This slowing of the entire contraction/relaxation cycle in cardiac myocytes is similar to the findings from the whole heart Langendorff experiments (Fig. 2 and Table 2). Overall, these data indicate cardiac myocytes from mice with tumors had significantly diminished contractile function and slowed relaxation.

### Cardiac myocyte calcium cycling

**Figure 6:**
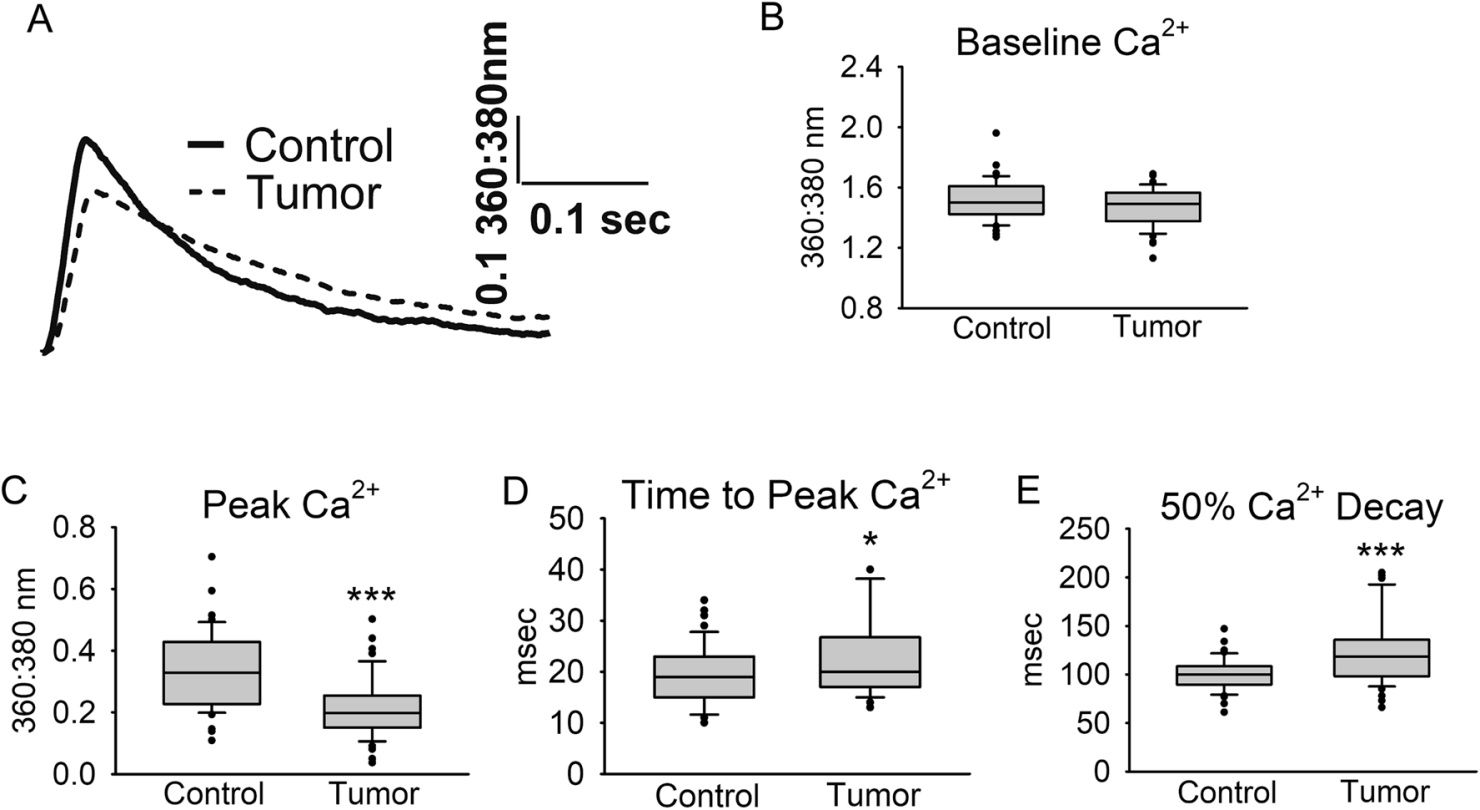
Cardiac myocyte calcium transient measurements. (**A**) Representative traces, (**B**) baseline calcium, (**C**) peak calcium, (**D**) time from baseline to peak calcium, and (**E**) time from peak to 50% calcium decay. Myocytes were paced at 0.5 Hz and 30 °C. N = 45–48 myocytes from 3 mice/group, data are presented as median + / − IQR. *P < 0.05, ***P < 0.001.

To determine whether impaired calcium cycling was contributing to cardiac myocyte functional deficits, calcium transients were measured using the calcium indicator dye Fura-2AM. Representative traces are illustrated in Fig. 6A. There was no difference in basal calcium levels (*P* = 0.08, Fig. 6B). Similar to the sarcomere length data, calcium transient peak height was decreased by 35% (*P* < 0.001) in myocytes from the Tumor group compared to the Control group (Fig. 6C). In addition, myocytes from the Tumor group displayed significant increases in time to peak calcium (Fig. 6D) and time from peak to 50% calcium decay (Fig. 6E). Together, these data show impaired calcium cycling evidenced by decreased calcium during contraction and slow reuptake during relaxation.

## Discussion

This study provides new understanding of the physiological mechanisms underlying cancer cachexia-induced cardiomyopathy. Cachexia markedly depresses heart performance in humans^12^ and in animal models^10,11^. However, it is unclear whether this is due to the systemic effects of cachexia or rather due to organ/cellular intrinsic functional deficits. Main new findings include marked contractile and relaxation deficits intrinsic to whole hearts and isolated adult cardiac myocytes. Reductions in the peak intracellular calcium amplitude and in the calcium transient decay rate in cardiac myocytes closely mirrored myocyte contraction/relaxation deficits, implicating a significant role for myocyte-intrinsic impaired calcium cycling in decreased cardiac function. This study provides the first data, to our knowledge, demonstrating cardiac myocyte intrinsic dysfunction in mice with overt tumor-induced cachexia, giving mechanistic insight into the underlying causes of in vivo cardiac dysfunction. Importantly, we demonstrate that cellular-level deficits in cardiac myocyte contractility and calcium handling critically underlie cancer cachexia-induced cardiomyopathy.

The presence of cardiac functional deficits intrinsic to the heart muscle and independent of the catabolic milieu of the diseased animal cannot be discerned from in vivo echocardiography experiments. Cachexia is a complex syndrome, with tumor- and host-derived soluble circulating signaling factors leading to a whole-body catabolic response characterized by systemic inflammation and significant metabolic perturbations affecting multiple tissues^36^. Multiple circulating cytokines^18,35,36^, alterations in β-adrenergic stimulation^37^, and decreased respiratory capacity^16^ in the in vivo environment may contribute directly and indirectly to the impaired cardiac function observed in vivo. Indeed, sympathoadrenal dysfunction resulting from altered autonomic nervous system activation may contribute to changes in heart rate and heart rate variability in humans with cachexia^38,39^. Atrophy of the diaphragm muscle^16^ coupled with anemia^38,40^ and altered metabolic substrate availability^41^ may impede energy production pathways in the heart. While many factors contribute to the overall decrease in cardiac function in cachexia, the present work distinguished isolated heart and myocyte-intrinsic dysfunction apart from the cachectic, in vivo environment as an additional key contributor to cachexia-induced cardiovascular insufficiency. Although systemic factors likely also lead to long-term changes in gene and protein expression that may affect ex vivo function, this study, by controlling the ex vivo environment, suggests that cardiac dysfunction is at least partially intrinsic to the cardiac myocyte and not simply an acute response to signaling from external stimuli. Furthermore, we have discovered significant impairment in relaxation function in both the heart muscle and isolated myocytes that has not been identified in echocardiographic studies in animals or humans with cachexia.

This study is the first, to our knowledge, to identify decreased peak calcium and slow calcium decay as mechanistic contributors to cardiac myocyte contraction and relaxation deficits in cachectic mice. The molecular underpinnings of impaired calcium cycling could involve multiple mechanisms. One possible mechanism is the alteration in the content of myocyte calcium handling proteins or by post-translational modifications of these proteins. In addition, it is appreciated that cardiac tissue is one of the most metabolically active tissues in the body, requiring a constant supply of energy in both active and resting states^42^. Here, both myofilament cross-bridge cycling and calcium cycling are major contributors to ATP utilization in heart muscle^43^. As cachexia is a syndrome characterized by a multitude of metabolic and energetic changes culminating in depleted energy stores^36^, it is reasonable to suggest that deficits in whole body metabolism and energy stores would in turn induce changes in cardiac energy availability and utilization critical to maintain normal calcium handling and cross-bridge cycling performance. Some evidence of morphological and functional changes in mitochondria from cachectic tissue support this hypothesis^11,16,17^. Other possibilities include cachexia-mediated alterations in sympatho-adrenal signaling critical to cardiac muscle performance^37–39^. Elucidating the precise mechanism(s) will require significant additional experimentation, and this will no doubt be an important area for future research.

Previous research related to cancer cachexia and the heart has largely focused on elucidating mechanisms of cardiac atrophy. Decreased sarcomeric protein expression, proteolysis, autophagy, and metabolic remodeling have been identified as potential contributors^10,14,15,18,35,44^. In contrast, relatively little work has been done to understand underlying contributors to functional changes in the heart. Increased fibrosis, reactivation of fetal genes including upregulation of β-myosin heavy chain (β-MHC)^11,14^, and changes in mitochondrial function^16,17^ have been suggested to have a role. While these changes may indeed alter function, in the present study we also identify significant deficits in cardiac myocyte calcium cycling, which closely mirror the decrease in contraction amplitude and slow relaxation. Our data suggest an important role for decreased peak calcium and slow calcium decay to affect myocyte contractility and relaxation on a beat-to-beat basis.

Like others, we also found evidence of fibrosis in cachectic hearts^11,15^. Quantification revealed a small but significant increase in total fibrotic area. Fibrosis may therefore be expected to contribute to impaired relaxation in Langendorff-perfused hearts, but the significant relaxation deficits found in whole hearts were also found in isolated myocytes, suggesting fibrosis is not the only contributor to slow relaxation. Our findings of decreased calcium reuptake during myocyte relaxation may likely be a more important contributor to impaired relaxation. In addition, we did not find detectable levels of cTnI in serum (data not shown), ruling out significant acute cardiac injury in whole heart dysfunction.

A decreased ratio of α/β-MHC chain found in previous studies of C26-induced cachexia has also been proposed to contribute to impaired cardiac function^11^, as decreased α/β-MHC has been implicated in human heart failure pathophysiology^45,46^. β-MHC has slower cross-bridge cycling kinetics, which leads to slower maximal shortening velocity and subsequent decreased contractility^47,48^. Notably, adenoviral gene transfer studies aimed at altering the α/β-MHC ratio in isolated myocytes from rats and rabbits found that contractility changes were calcium independent^47,48^. In contrast, the present work shows decreased contraction and slowed relaxation closely mirror the decreases in calcium peak and slowed decay, suggesting that impaired intracellular calcium cycling has a predominant role in cachexia-induced contractile dysfunction. Although it is likely that increased β-MHC has some effect on cardiac function as well, the relative contributions of calcium cycling deficits and MHC isoform switching to overall function cannot be quantified from the current experiments. Importantly, we uncover calcium mishandling as at least partially causal for cardiac myocyte contractility deficits.

The field of cardio-oncology has become increasingly important in recent years due to the known cardiotoxic effects of many cancer therapies and increasing awareness of direct tumor effects on the heart^1,49^. Less is understood about the specific effects of cachexia on the heart aside from significant heart muscle atrophy in both animal models and humans^10^. As tumor effects and cachexia effects on the heart are occurring simultaneously, it is difficult to isolate the independent effects of cachexia. However, previous work showed that a similar model using female mice bearing C26 tumors exhibited relatively little body weight loss and cardiac atrophy after 21 days with tumors^20,50,51^. Despite decreased fractional shortening in vivo, myocytes from these animals had no change in contraction amplitude compared to controls, and inconsistency between studies in the time required to complete a contraction/relaxation cycle. These data, together with the data presented here, suggest a specific role for cachexia, independent of the presence of the tumor, in impaired function of the heart muscle and cardiac myocytes.

## Conclusions

In conclusion, we provide new evidence of significant contraction and relaxation deficits in Langendorff-perfused hearts and isolated cardiac myocytes from mice with cancer-induced cachexia. Furthermore, decreased peak calcium and transient decay rate in cardiac myocytes were shown to underlie functional impairments. Importantly, we elucidate intrinsic functional impairment in isolated heart muscle apart from other in vivo factors such as changes in energy availability and host- and tumor-derived signaling. These data provide mechanistic insight into the cell intrinsic basis of previous echocardiographic studies reporting in vivo cardiac dysfunction in animals and humans with cancer cachexia. Ultimately, the present work sheds new light on the effects of cachexia not only on cardiac atrophy, but also on heart muscle function. Because cardiac insufficiency can directly contribute to weakness, fatigue, and muscle wasting^21^, cardiac dysfunction may exacerbate the global effects of cancer-induced cachexia, creating a vicious cycle that increases cachexia severity and worsens patient outcomes^22^. Continued investigation of mechanisms contributing to the development and progression of cardiac functional deficits is necessary for the optimization of treatments and clinical care to increase quality and length of life for patients suffering from cancer-induced cachexia^1^.
